# Supplementary material for: Nuclear gene proximity and protein interactions shape transcript covariations in mammalian single cells
Source: Nat Commun. 2020 Oct 28;11:5445. doi: 10.1038/s41467-020-19011-5 (PMC7595044; doi:10.1038/s41467-020-19011-5)
Supplement: Supplementary file 1 — Supplementary Information [file 41467_2020_19011_MOESM1_ESM.pdf]

Supplementary Figures

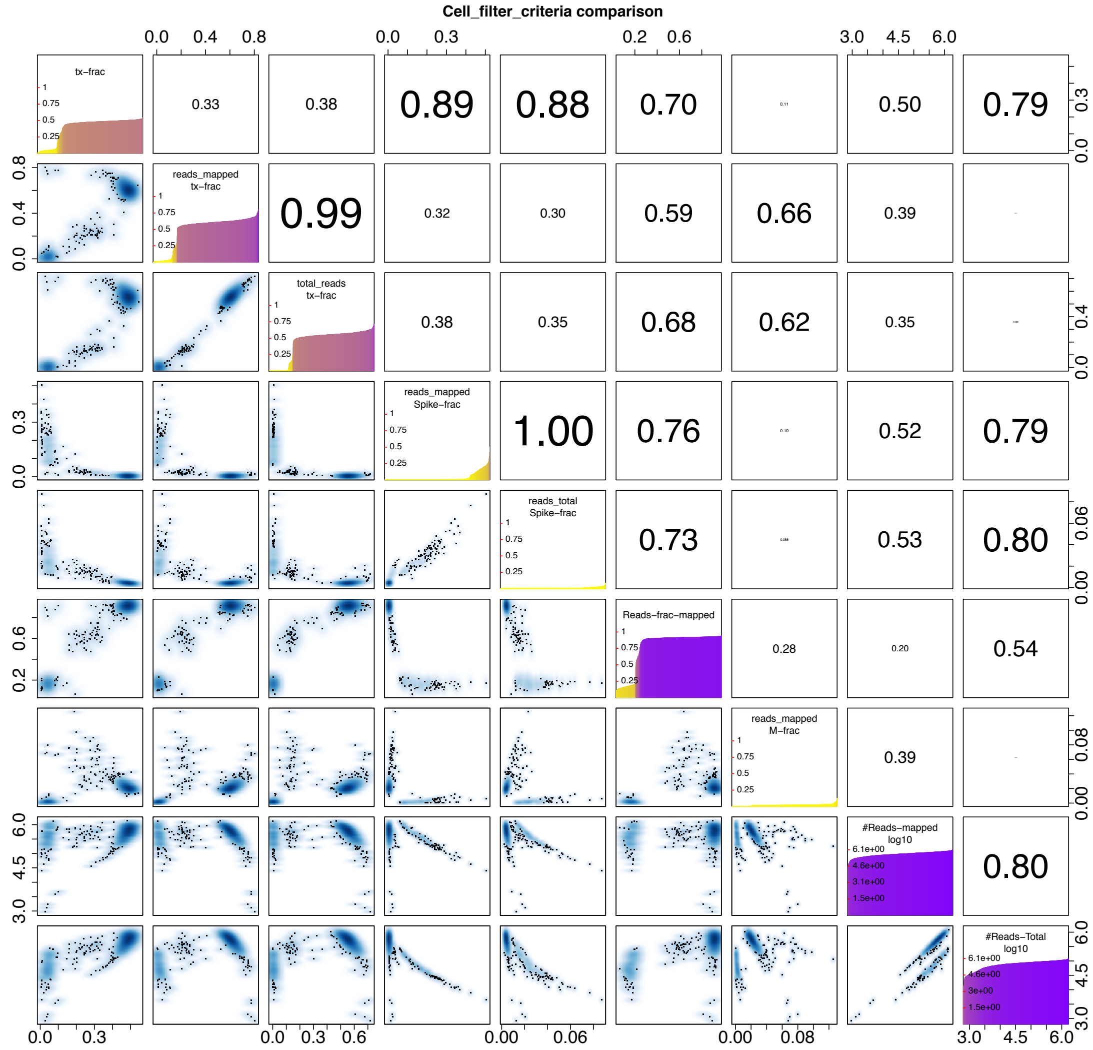

Supplementary figure 1 – mapping statistics for sequence quality control

Comparison of different read mapping statistics for identification of outlier cells. The diagonal shows the distribution of the respective measure over all cells. The lower triangle compares the respective measures as x- and y-values. The upper triangle shows the corresponding Spearman's correlation coefficients for these comparisons.

Metrics: (1) fraction of transcriptome with at least one mapped read, (2) fraction of mapped reads mapping to transcriptome, (3) fraction of total reads mapping to transcriptome, (4) fraction of mapped reads mapping to spike-ins, (5) fraction of total reads mapping to spike-ins, (6) fraction of reads mapping to genome or transcriptome, (7) fraction of reads mapping to mitochondrial genes or genome, (8) log10 total reads mapping to genome or transcriptome, (9) log10 total reads.

More information on this data representation is available in the online documentation of the *pairs* function in R.

Overall correlation between gene pairs varies depending on normalization

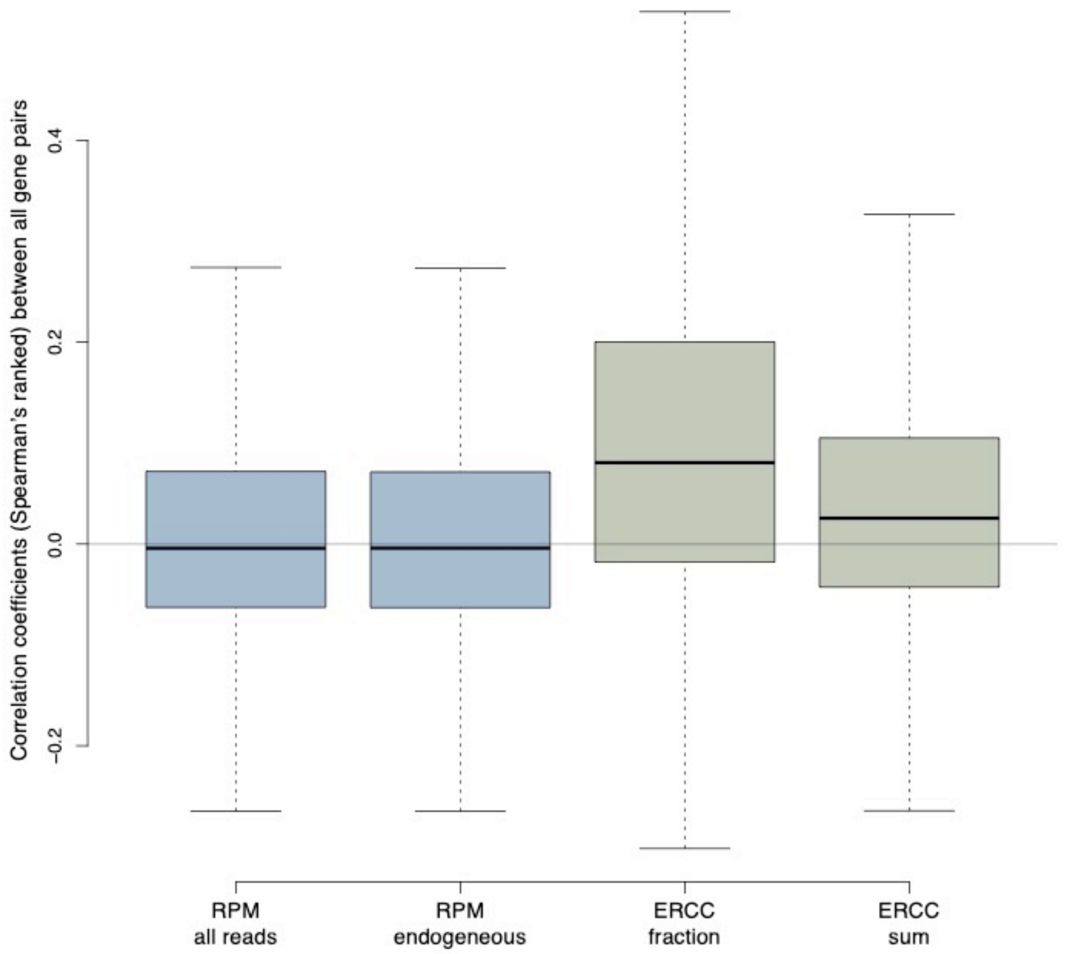

Supplementary figure 2 – comparison of normalization methods

Boxplot of all gene pair expression correlation coefficients after four different normalizations. Correlations were calculated for all 8989 genes over 108 cells (plate 1) resulting in 40.396.566 unique pairwise-correlations which were subsampled to 100.000 for visualization. Boxes indicate the 25 and 75 percentile. Whiskers indicate 1.5× interquartile range (IQR).

Deviations from the base line (y=0) indicate a possible global factor that artifically induces gene pair expression covariance due to incomplete normalization. RPM normalizations outperformes normalization by ERCC spike-in total reads and fraction.

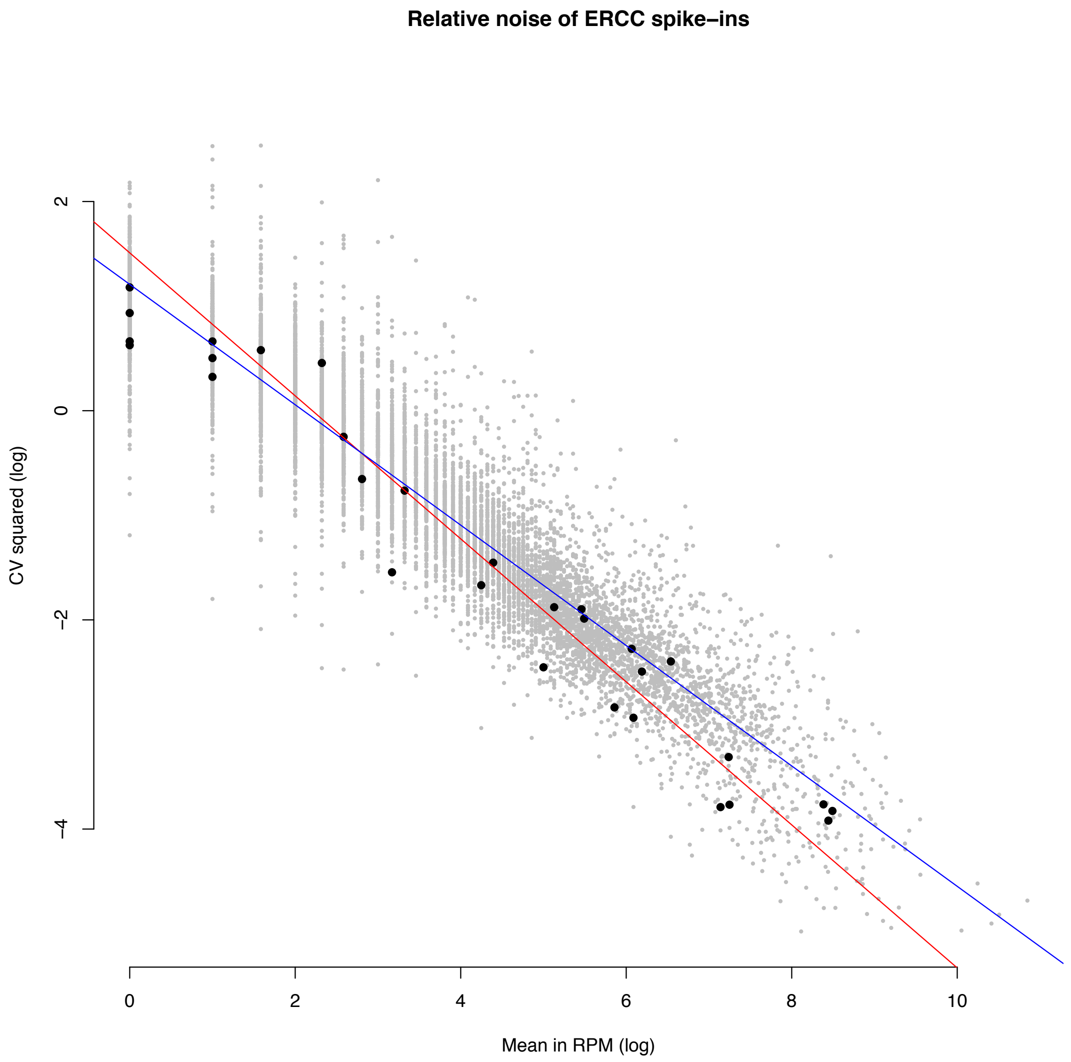

Supplementary figure 3 – relationship between gene expression median and variation

Gene expression variation (here estimated as log2 of the coefficient of variation squared) is inherently linked to the median expression (here shown as log2 of RPM). They are know to be linearly depenent in log-space.

Spike-ins are highlighted as black dots. Blue line shows the least-squared fit of expression variation to expression median for endogeneous transcripts, the red line shows the fit for spike-ins. Notably, the variation of endogeneous transcripts exceeds the technical variation measure by ERCC spike-ins already at around eight reads per million.

For this plot the expression data was normlized to the fraction of ERCC-spike-ins to preserve differences due to cell size variations. These variations are relevant for for estimation of endogeneous gene expression variation but can be considered a confounding factor in gene co-expression (covariance) analysis.

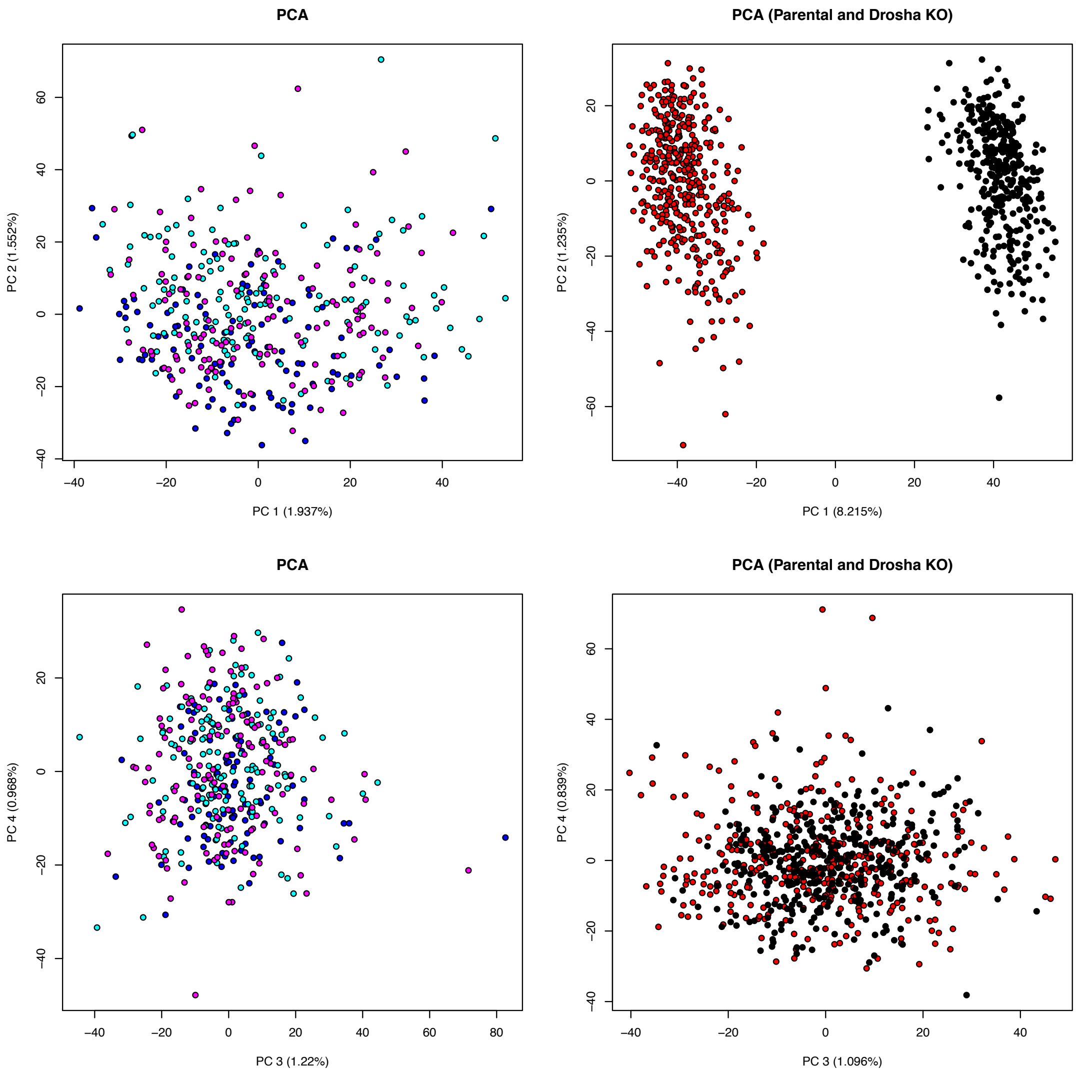

(Figure continues on next page)

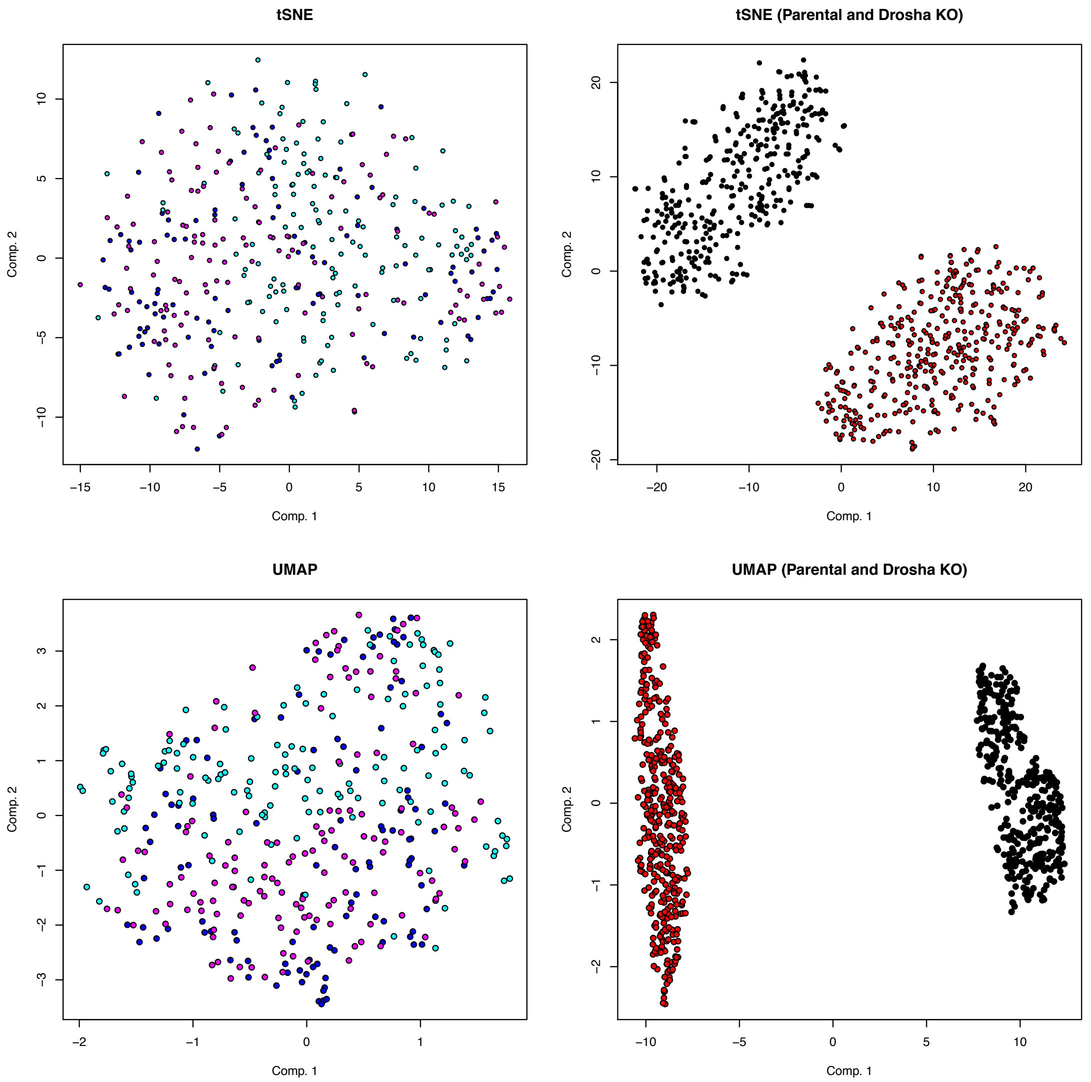

Supplementary figure 4 – dimensionality reduction

Dimensionality reduction of the cells considered in this study. Left column: Only parental cells, colors indicate the three sequencing runs. Right column: Parental cells (black) and Drosha KO cells (red). First row: PCA components 1 and 2. Second row: PCA components 3 and 4. Third row: tSNE components 1 and 2. Fourth row: UMAP components 1 and 2.

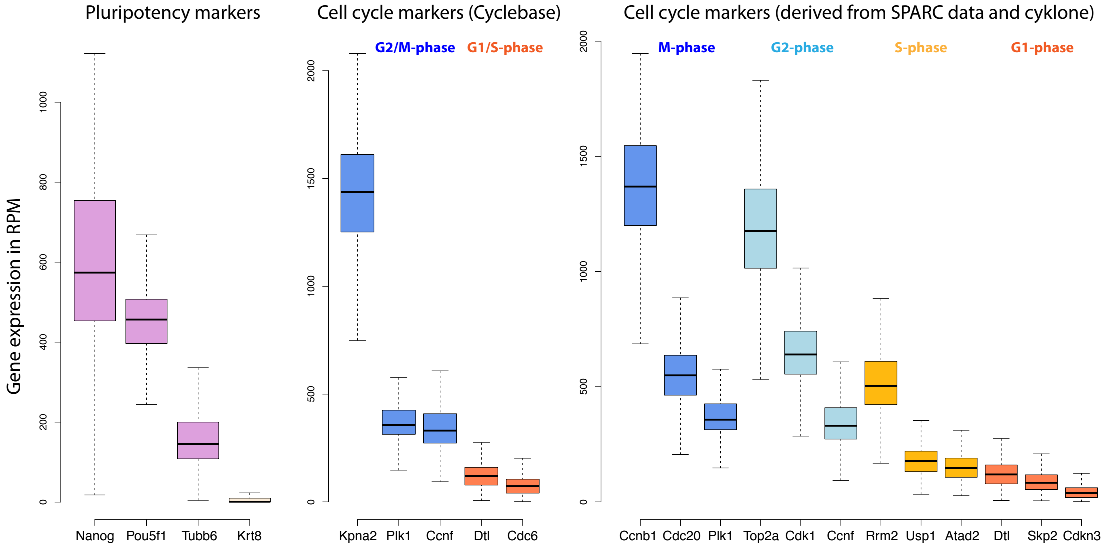

Supplementary figure 5 – Marker gene expression confirms pluripotency and validates G2M-phase sorting

Expression of selected marker genes in RPM in 355 control cells. Boxes indicate the 25 and 75 percentile. Whiskers indicate 1.5×IQR.

Left panel: Nanog and Pou5f1 (Oct4) are pluripotency markers. Nanog is known to be highly variable between cells. Tubb6 is a marker that is highly expressed in serum and lowly expressed in 2i+LIF medium. Krt8 is a differentiation marker.

Central panel: Cell cycle markers for human from Cyclebase. Kpna2 is a G2/M-phase marker. Plk1 and Ccnf are makers for M- and G2 phase respectively. Dtl expression marks G1-phase and Cdc6 has peak expression in G1/S-phase.

Right panel: In-house cell cycle markers derived from hESC single-cell RNA and protein expression and cell cycle assignement using the *cyclone* function of the R package *SCRAN*.

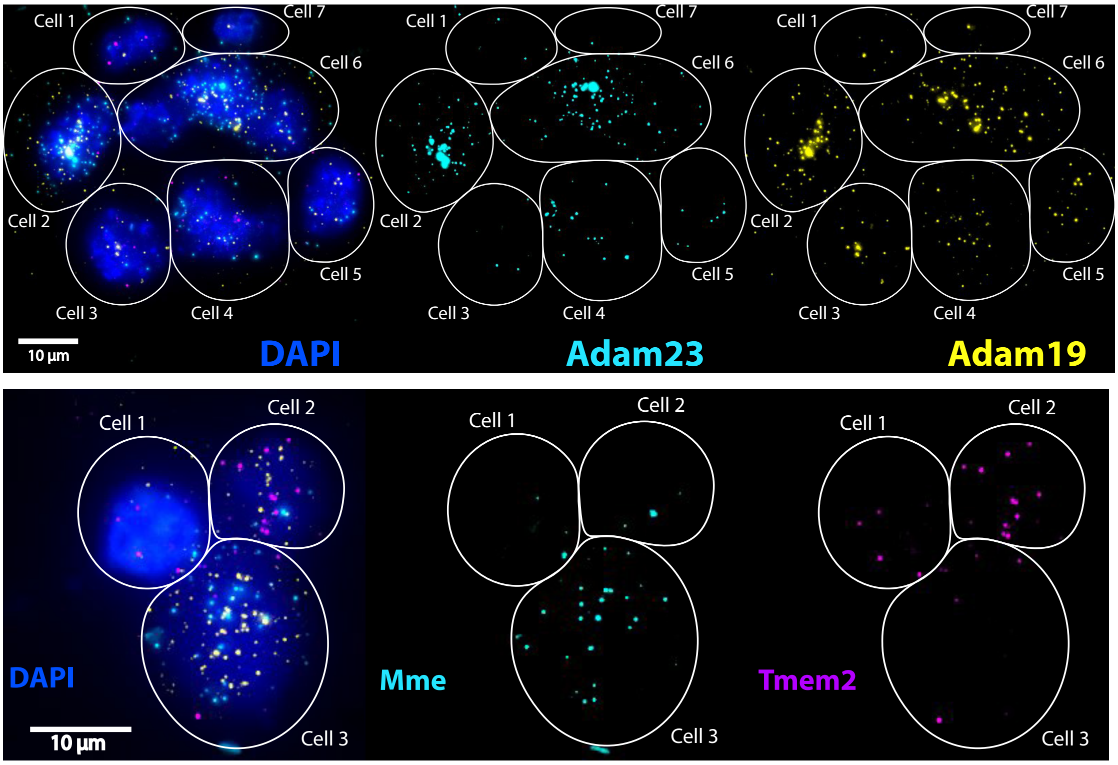

Supplementary figure 6 – validation of covariance patterns by smFISH I

Upper panel: Example of cells (prental, WT) that show similar expression levels of Adam23 and Adam19 (high: cells 2, 6; medium: cells 4, 5; low: cells 1, 3, 7). Lower panel: Example of cells (Drosha KO) that show inverse expression levels of Mme and Tmem2 (cell 3: high Mme, low Tmem2; cells 1, 2: low Mme, high Tmem2). Cell outlines were drawn manually for illustration. These images are examples for visualization only. Cell outlines are hand-drawn. A full quantification using a grid approach and Matlab software was performed and the results are presented in supplementary figure 7 (below).

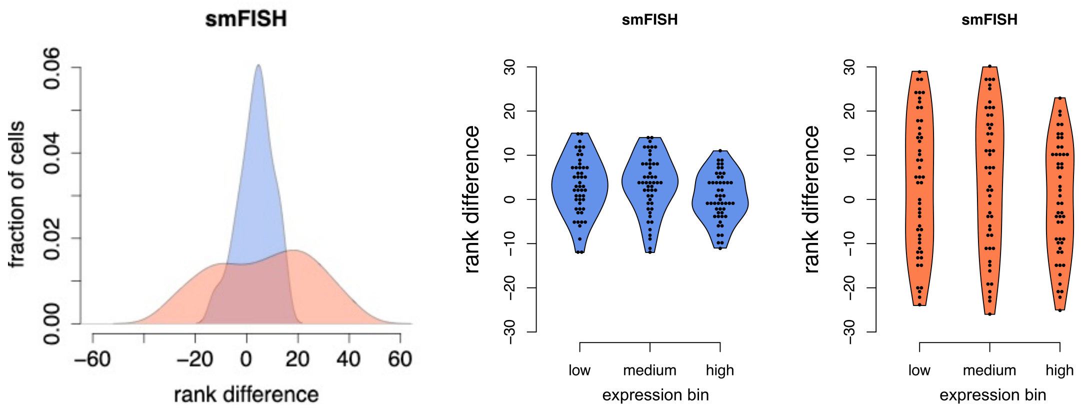

Supplementary figure 7 – validation of covariance patterns by smFISH II

Left panel: Positively covarying genes (Adam19 and Adam23, blue area) show little variation in the difference of expression rank, indicating that they are commonly expressed together in single cells at similar expression ratios. For this cells got assigned values according to the order of expression of the respective gene, e.g. the cell with the highest expression being assigned 1. Negatively covarying genes (Mme and Tmem2, orange area) show a bimodal distribution of rank differences, indicating the presence of 2 cell populations, one with high abundances of gene A and low abundances of gene B, and one with high abundances of gene B and low abundances of gene A. Right panel: Same data as in the left panel but stratified into expression bins and represented as combined violin and bee swarm plots. The low expression bins was defined as the 50 cells with lowest expression. Medium and high expression bins were subsequently the next 50 cells with higher expression. Ranks were assigned within each bin. Positively covarying genes (blue) show little variation in expression rank differences while negatively covarying genes pairs (orange) show bimodality.

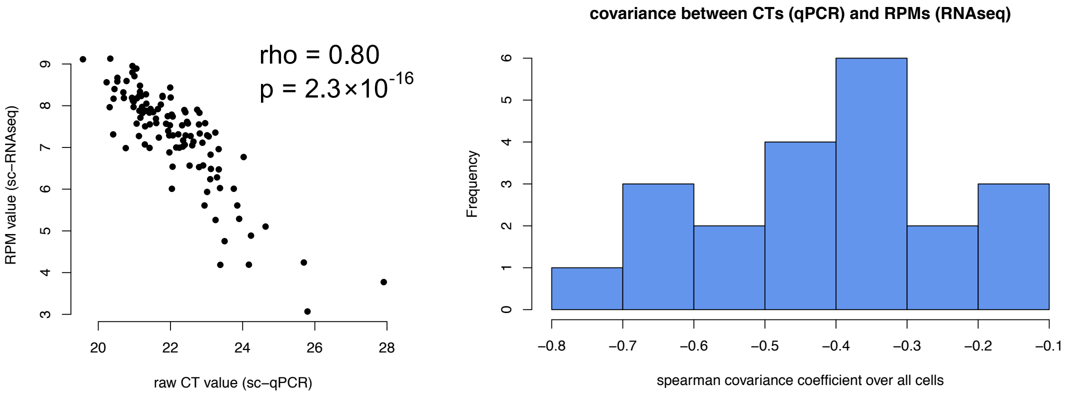

Supplementary figure 8 – validation of covariance patterns by sc-qPCR

Left panel: CT values from sc-qPCR are almost perfectly anti-correlated to RPM values from scRNAseq for the Utf1 gene. Right panel: Correlation coefficients (rho-values) between CT values and RPM values for all 21 genes that have been detected in the sc-qPCR. All coefficients are negative with an average smaller than -0.4.

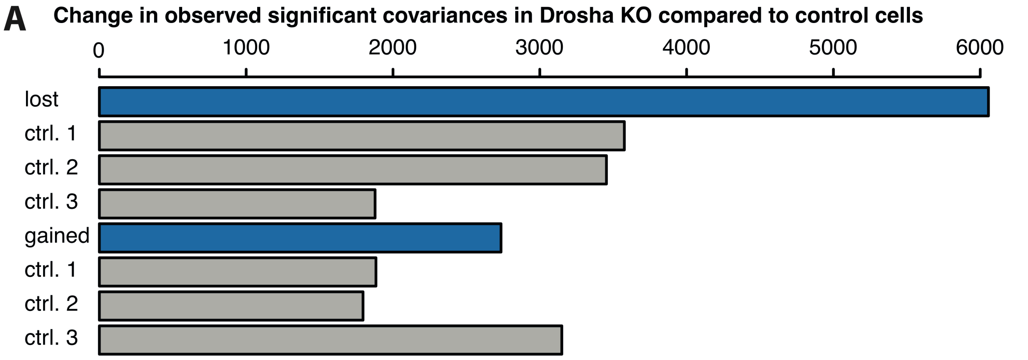

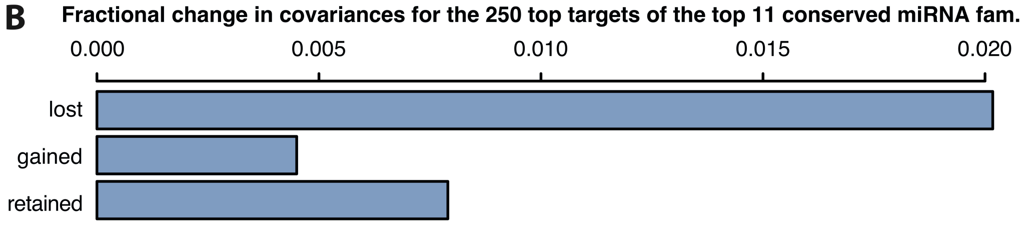

C

Network properties	Positive covariances		Negative covariances	
	Control cells	Drosha KO	Control cells	Drosha KO
Cluster coefficient	0.23	0.57	$1.24{*}10^{-5}$	$0.75{*}10^{-5}$
Average cluster coefficient	0.19	0.18	$3.32{*}10^{-5}$	$0.46{*}10^{-5}$
Diameter	13	15	12	14
Centrality	0.98	0.98	0.98	0.98
Average path length	4.24	4.82	4.23	4.48
Average degree	10.07	8.41	5.74	4.33
Isolated nodes	2265	2866	3255	4017

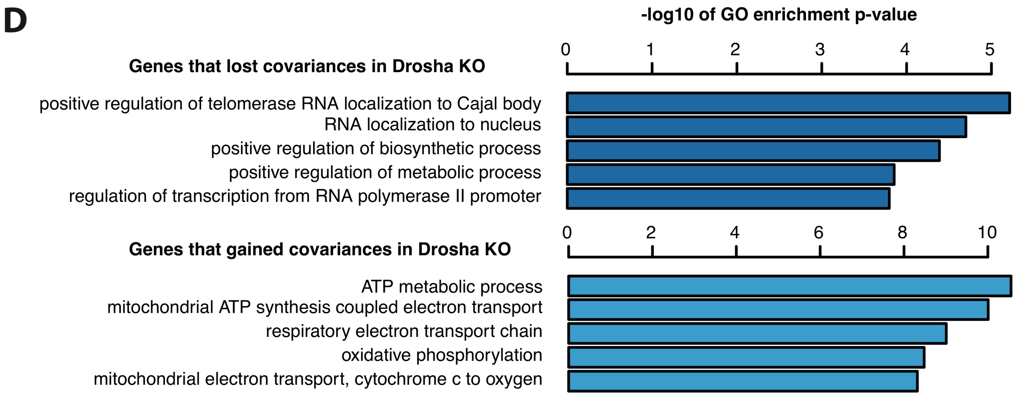

Supplementary figure 9 – change in network properties in Drosha KO

(Figure legend on next page)

Drosha$^{-/-}$ (KO) cells are void of all canonical miRNAs. Their co-expression network properties differ from those of control cells.

A) Comparison of total number of covariances that are unique to Drosha KO (*gained* in Drosha KO in comparison to control cells) or unique to control cells (*lost* in Drosha KO in comparison to control cells). Drosha KO cells mainly lose significant covariances. Covariances that are gained are comparable to permuted controls. Permuted controls were generated shuffling covariance gene pair assignment for the one of the replicates randomly.

B) Gene pairs that are targeted by the same miRNA specifically lose more covariances than they gain or retain in the Drosha KO.

C) Gene co-expression network properties. Cluster coefficient describes the overall connectivity of the network. The average of this coefficient describes the mean connectivity of each individual node. The diameter describes the shortest past between the most distand nodes in the network. The centrality is a measure for whether the network is hierarchical with central hubs. The average path length describes the mean number of edges that have to be traversed to connect two nodes. The average degree decribes how many neighbors a representative node has. Finally, the number of isolated nodes describes the number of nodes wich have no connections to other nodes.

D) Gene ontology enrichments of genes that lose or gain (see subfigure A) covariances in Drosha KO cells in comparison to control cells (p-values represent Fisher's exact test as impemented in TopGO).

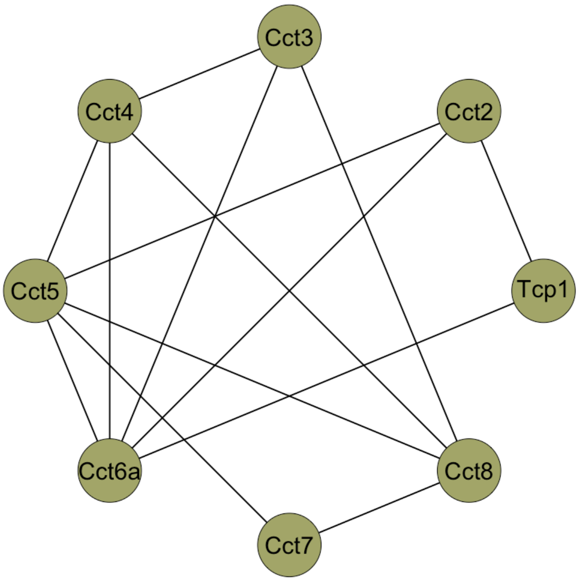

Gene name	Location
Tcp1	chr17
Cct2	chr10
Cct3	chr3
Cct4	chr11
Cct5	chr15
Cct6a	chr5
Cct7	chr6
Cct8	chr16

Supplementary figure 10 – Covariances between genes involved in the TCP1-complex

Gene co-expression network of genes involved in forming the T-complex 1. Significant covariances are indicated with black lines. Theses genes are scattered throughout the genome as shown in the table on the right.

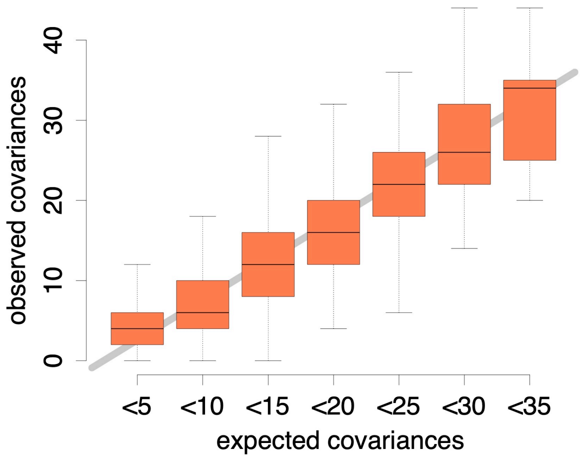

Supplementary figure 11 – covariance enrichment score proof of priciple

Random gene sets of 100 genes each have been binned according to the number of expected covariations predicted by our background model. Number of observed covariations in each bin are shown (N=987, 4416, 1173, 405, 298, 82, and 22 respectively). Boxes indicate the 25 and 75 percentile. Whiskers indicate 1.5×IQR. There is a good fit between predicted and observed covariations for thousands of random gene sets.

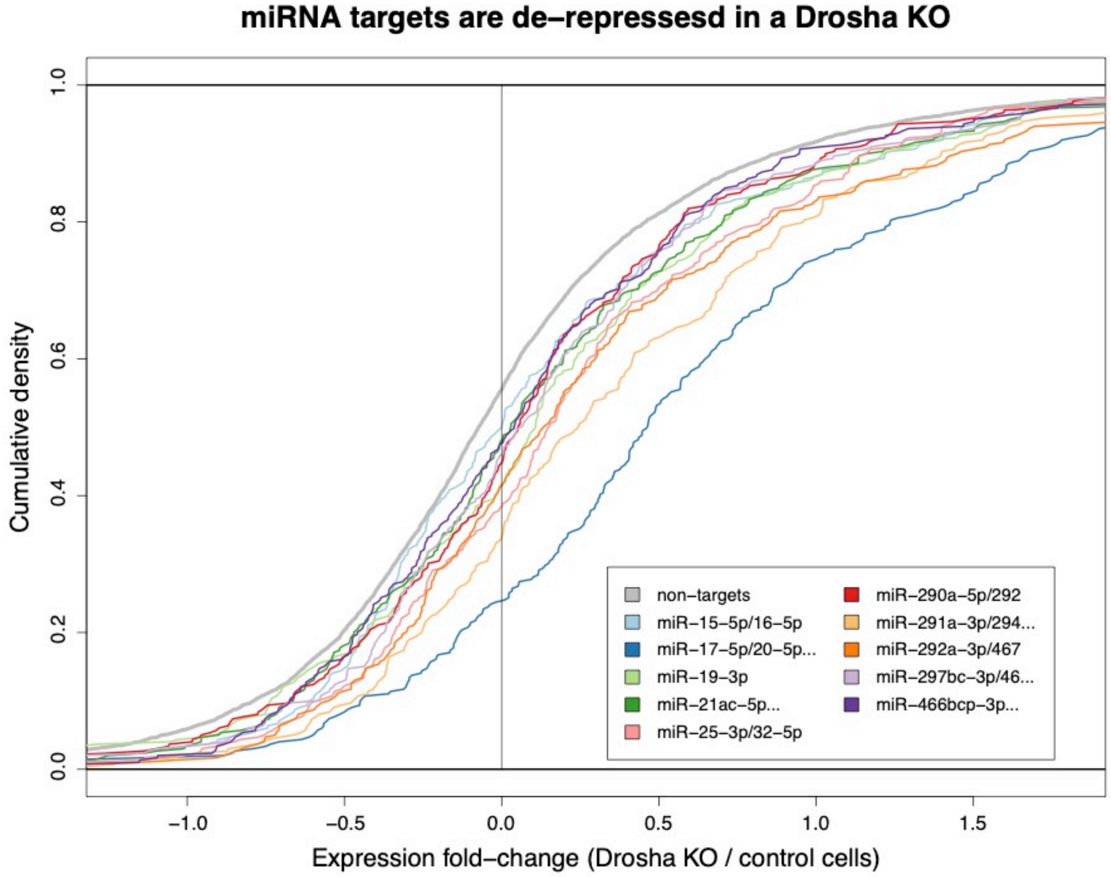

Supplementary figure 12 – CDFs of miRNA target fold-changes

Cumulative distribution function of expression fold-changes between parental cells and Drosha KO cells (void of miRNA) for the top 300 targets of the most abundant miRNA in mESC and genes that are not predicted to be targets of any of these miRNA.

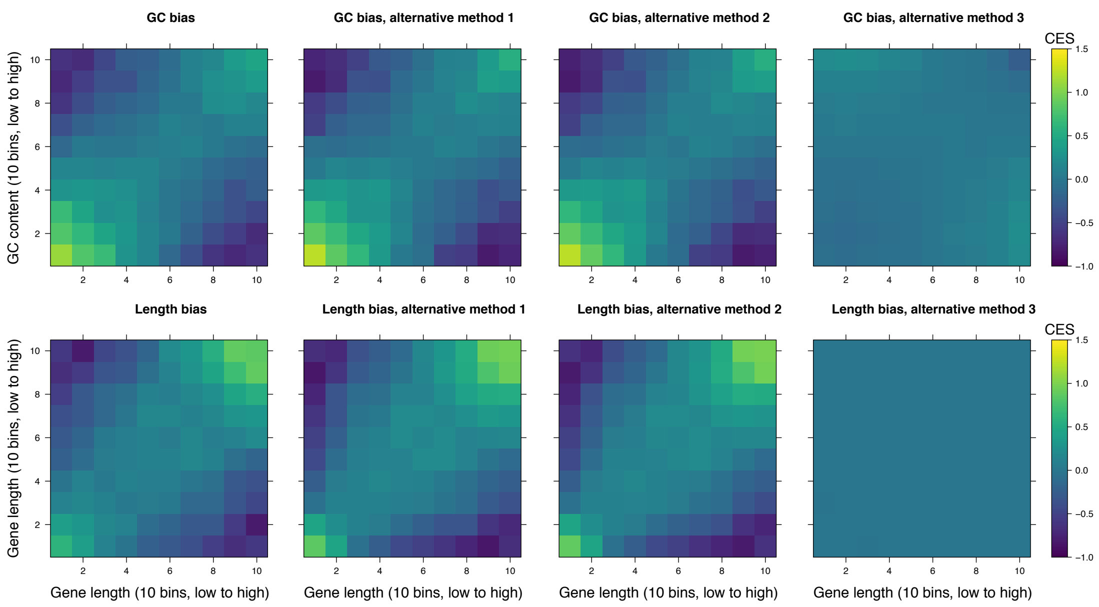

Supplementary figure 13 – GC and length biases

Heat maps of co-expression enrichment scores (CES) in 10 bins of genes according to GC content or length respectively. Genes with similar GC content of length show enrichment for co-expression. Alternative method 1: Mapping with STAR, no de-duplication, scran normalization using size factors. Alternative method 2: Same as method 1 but with additional length normalization. Alternative method 3: Same as method 2 but with hard correction of GC content and length biases in CES background model.

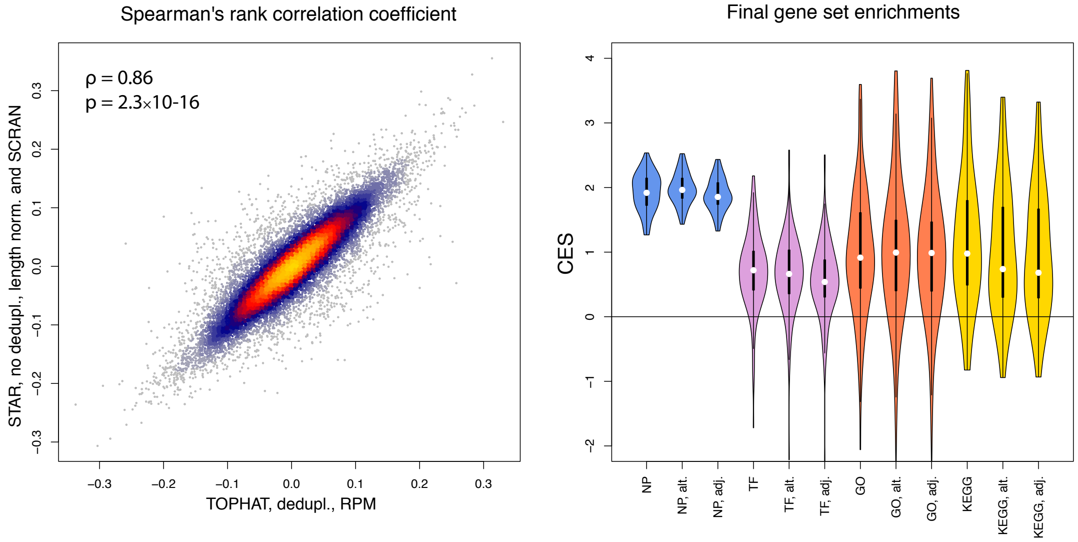

Supplementary figure 14 – Robustness of correlations and CES

Left panel: Heat scatter plot of Spearman's rank coefficients for pair-wise correlations of 200 randomly sampled genes using two different analysis pipelines. Pipeline one uses TOPHAT for mapping, applies de-duplication and an RPM normalization. Pipeline two uses STAR for mapping, applies no de-duplication and uses a length normalization in combination with scran's estimation of size factors. There is good agreement between the correlation coefficients. **Right panel**: Co-expression enrichment scores (CES) for top 50.000 nuclear proximal gene-pairs (purple), top 50 transcription factor targets (blue) and all GO (orange) and KEGG pathway (yellow) annotation. First violin in each group shows original analysis (TOPHAT, de-dupl., RPM), second violin shows suggested analysis (STAR, no de-dupl., length and scran normalization), third violin shows additional hard correction of GC and length biases in the CES background model. Overall, co-expression enrichments were retained in a similar magnitude independent of exact analysis pipeline and GC/length bias correction.

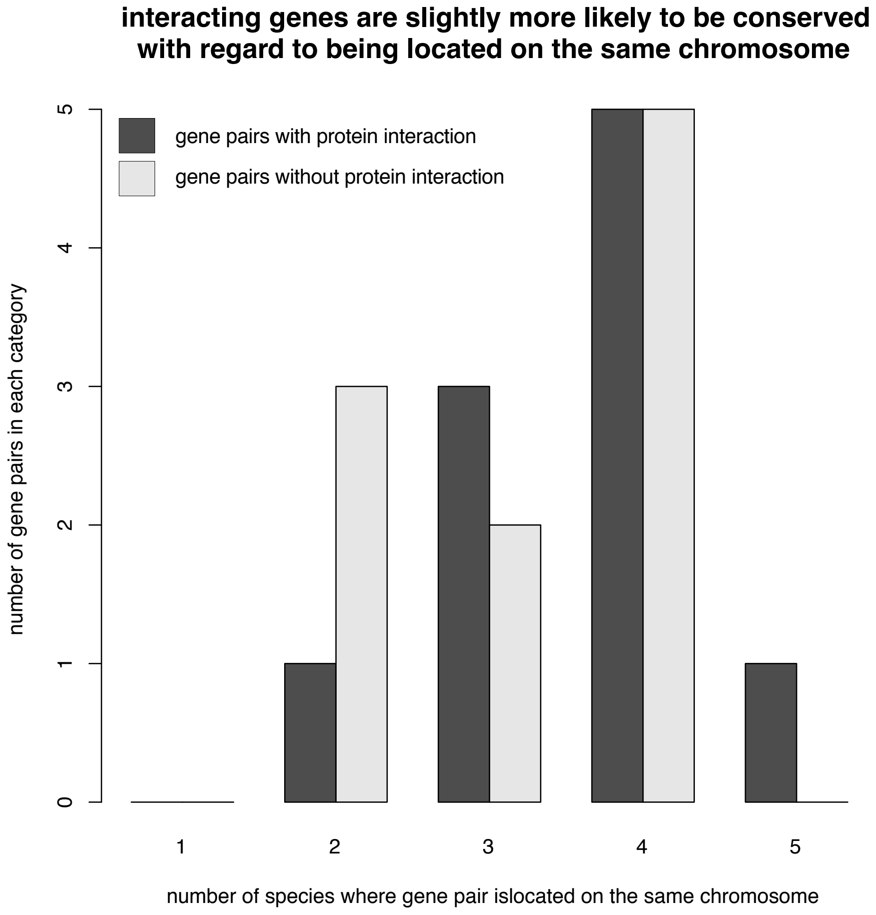

Supplementary figure 15 – Co-localization of gene pairs in distant species

Co-localization of covarying gene pairs in five model organisms (human, mouse, rat, clawed frog and zebrafish). A subset (N=10) of gene pairs interacting on the protein level (dark grey) is compared to a subset (N=10) of genes not interacting on the protein level (light grey).

Supplementary Tables

Supplementary table 1 – Mapping statistics across the three replicates

Measurement	Replicate	Min.	25%	Median	75%	Max.
Total mapped reads	1	136638	275756	349539	430778	551584
	2	129862	225867	269602	313955	417829
	3	130947	296490	374160	425664	587444
Percentage of ERCC spike-in reads	1	0.45	0.66	0.72	0.78	1.16
	2	0.35	0.67	0.74	0.81	1.17
	3	0.38	0.65	0.72	0.78	1.10

Supplementary table 2 – Cell filtering

Remaining cells considered for analyses after various filtering steps.

Number of cells per plate after filtering steps	Plate 1	Plate 2	Plate 3
All wells	384	384	384
Remove intentionally empty wells	378	378	378
QC based on mapping statistics and PCA	189	284	288
Cell cycle filtering with SCRAN	179	257	262
Of those control [Ctrl]	**107**	**124**	**124**
Of those Drosha$^{-/-}$ [KO]	72	133	138

Supplementary table 3 – Gene filtering

Remaining genes considered for analyses after various filtering steps.

Number of genes after various filtering steps	Ctrl	KO	overlap
Before filtering	19,127	19,127	19,127
Median expression >0 RPM in all three plates	**8989**	9105	8501
Mean expression >16 RPM in all 3 plates	6687	6873	6202
Overlap of expression filters	6687	6873	6202
Median expression >0 RPM in all three plates and no ribosomal prot.	8763	8877	8280

Supplementary table 4 – Total numbers of significant covariances

Absolute numbers of significant covariances (p-value estimation for Spearman's ranked see Methods; p-value applied in each replicate was 0.01 or 0.05 respectively) between individual genes for different data subsets as well as their integration. False discovery rate (FDR) was estimated as ratio of the maximal number of significant covariances in 100 permutations of the gene expression matrices compared to the actual number of observed significant covariances.

Number of significant covariances	Positive ($p<0.01$)	Negative ($p<0.01$)	Positive ($p<0.05$)	Negative ($p<0.05$)
Control cells	52,695	29,125	221,382	165,612
Control cells, riboproteins excluded	**42,938**	**24,390**	202,581	142,265
Drosha KO cells	38,270	19,716	164,518	126,436
Drosha KO cells, riboproteins excluded	27,147	14,115	98,673	145,723
Estimated FDR	2,046 / (42,938 + 24,390) = 0.0304			

Supplementary table 5 – Covariances between pluripotency genes

Significant covariances of and between important pluripotency factors. The last two rows show genes that have not been previously described to have functions in defining pluripotency.

Sign. cov.	Esrrb	Nanog	Pou5f1	Sox2	Klf4	Zfp42
Total number of sign. pos. cov.	97	233	68	48	62	16
Total number of sign. neg. cov.	121	221	47	31	59	13
Pos. cov. examples	Sox2, Klf4	Klf4		Esrrb, Klf4	Esrrb, Sox2, Nanog	
Neg. cov. examples		Pou5f1	Nanog			
Novel pos. examples	Cd9	Cd9	Dnmt3b, Wdr74	Cd9	Cd9	Cd9
Novel neg. examples	Dnmt3b, Wdr74	Dnmt3b, Wdr74	Cd9	Dnmt3b, Wdr74	Dnmt3b, Wdr74	Dnmt3b, Wdr74
