# Supplementary material for: Nuclear gene proximity and protein interactions shape transcript covariations in mammalian single cells
Source: Nat Commun. 2020 Oct 28;11:5445. doi: 10.1038/s41467-020-19011-5 (PMC7595044; doi:10.1038/s41467-020-19011-5)
Supplement: Supplementary file 3 — Reporting Summary [file 41467_2020_19011_MOESM3_ESM.pdf]

nature research

Corresponding author(s): Marc R. Friedländer

Last updated by author(s): Aug 31, 2020

Reporting Summary

Nature Research wishes to improve the reproducibility of the work that we publish. This form provides structure for consistency and transparency in reporting. For further information on Nature Research policies, see Authors & Referees and the Editorial Policy Checklist.

Statistics

For all statistical analyses, confirm that the following items are present in the figure legend, table legend, main text, or Methods section.

n/a	Confirmed	
☐	☒	The exact sample size (*n*) for each experimental group/condition, given as a discrete number and unit of measurement
☐	☒	A statement on whether measurements were taken from distinct samples or whether the same sample was measured repeatedly
☐	☒	The statistical test(s) used AND whether they are one- or two-sided<br>*Only common tests should be described solely by name; describe more complex techniques in the Methods section.*
☒	☐	A description of all covariates tested
☒	☐	A description of any assumptions or corrections, such as tests of normality and adjustment for multiple comparisons
☒	☐	A full description of the statistical parameters including central tendency (e.g. means) or other basic estimates (e.g. regression coefficient) AND variation (e.g. standard deviation) or associated estimates of uncertainty (e.g. confidence intervals)
☐	☒	For null hypothesis testing, the test statistic (e.g. *F*, *t*, *r*) with confidence intervals, effect sizes, degrees of freedom and *P* value noted<br>*Give P values as exact values whenever suitable.*
☒	☐	For Bayesian analysis, information on the choice of priors and Markov chain Monte Carlo settings
☒	☐	For hierarchical and complex designs, identification of the appropriate level for tests and full reporting of outcomes
☒	☐	Estimates of effect sizes (e.g. Cohen's *d*, Pearson's *r*), indicating how they were calculated

Our web collection on statistics for biologists contains articles on many of the points above.

Software and code

Policy information about availability of computer code

Data collection	No software was used for data collection.
Data analysis	tophat/2.0.12<br>bowtie2/2.2.3<br>samtools/0.1.18<br>custom script "exonize.pl" for counting of reads mapped per gene<br>iFISH v2.0.3 (http://ifish4u.org/) and MatLab R2017b was used for the FISH analysis

For manuscripts utilizing custom algorithms or software that are central to the research but not yet described in published literature, software must be made available to editors/reviewers. We strongly encourage code deposition in a community repository (e.g. GitHub). See the Nature Research guidelines for submitting code & software for further information.

Data

Policy information about availability of data

All manuscripts must include a data availability statement. This statement should provide the following information, where applicable:
- Accession codes, unique identifiers, or web links for publicly available datasets
- A list of figures that have associated raw data
- A description of any restrictions on data availability

BioProject: PRJNA592852
SRA submission: SUB6625581
STRING DB dataset: http://version10.string-db.org/download/protein.links.detailed.v10/10090.protein.links.detailed.v10.txt.gz
Gene Ontology analysis: http://www.informatics.jax.org/faq/GO_dload.shtml

Field-specific reporting

Please select the one below that is the best fit for your research. If you are not sure, read the appropriate sections before making your selection.

☒ Life sciences ☐ Behavioural & social sciences ☐ Ecological, evolutionary & environmental sciences

For a reference copy of the document with all sections, see nature.com/documents/nr-reporting-summary-flat.pdf

Life sciences study design

All studies must disclose on these points even when the disclosure is negative.

Sample size	Sample size was chosen based on the single cell technology available. Smart-seq2 was run on three 384 well plates. We decided on this format because the plate sizes are fixed from the company and three plates seemed feasible in terms of work effort with labor-intensive cell work and also in terms of costs. In fact this design turned out to give us excellent statistical strength. Each plate was split between control cells and Drosha KO cells. Smart-seq2 is highly quantitative and we expect to get highly quantitative covariance measures with around 100 cells per plate and condition.
Data exclusions	Cells were excluded based on mapping statistics, PCA and cell cycle assignment. This was to exclude duplets (a common issue in single cell sequencing) and empty wells, as well as cells that were in the wrong cell cycle stage despite FACS. These criteria were not pre-determined, rather we performed various analysis to detect outlier cells. Since single-cell sequencing data was quite state-of-the-art when we designed the study, we learned how to analyze the data after it was generated.
Replication	Three sequencing runs were analysed separately. All replicates were then included in the analysis. All replicates were successful, none were omitted from the final study.
Randomization	NA (For sampling in permuted controls random sampling was applied.) It is not clear to us how our experiment with mammalian single cells could have been performed with randomization.
Blinding	NA (We worked with highly homogeneous cells. Only one different condition was tested as validation experiment.)

Reporting for specific materials, systems and methods

We require information from authors about some types of materials, experimental systems and methods used in many studies. Here, indicate whether each material, system or method listed is relevant to your study. If you are not sure if a list item applies to your research, read the appropriate section before selecting a response.

Materials & experimental systems

n/a	Involved in the study	
☒	☐	Antibodies
☐	☒	Eukaryotic cell lines
☒	☐	Palaeontology
☒	☐	Animals and other organisms
☒	☐	Human research participants
☒	☐	Clinical data

Methods

n/a	Involved in the study	
☒	☐	ChIP-seq
☒	☐	Flow cytometry
☒	☐	MRI-based neuroimaging

Eukaryotic cell lines

Policy information about cell lines

Cell line source(s)	The DroshaF E14 129Sv-derived mouse embryonic stem cell line (mESC) was provided by M. Chong (Chong et al, 2008).
Authentication	Drosha KO was validated via qPCR for Drosha and via sRNA-seq (Bonath et al, 2018, study performed in our group on the exact cell batch used in this present study). Drosha KO were void of canonical miRNA. qPCR and sequencing are both established methods for authenticating the molecular identity of cell lines.
Mycoplasma contamination	Cells were specifically tested for Mycoplasma contamination (see Online Methods). The tests revealed that there was ni Mycoplasma contamination.
Commonly misidentified lines (See ICLAC register)	We have not worked with any commonly misidentified cell lines.
