# Supplementary material for: Nuclear gene proximity and protein interactions shape transcript covariations in mammalian single cells
Source: Nat Commun. 2020 Oct 28;11:5445. doi: 10.1038/s41467-020-19011-5 (PMC7595044; doi:10.1038/s41467-020-19011-5)
Supplement: Supplementary file 4 — Description of Additional Supplementary Files [file 41467_2020_19011_MOESM4_ESM.pdf]

Description of Additional Supplementary Files

File Name: Supplementary Data 1
Description: Gene expression values in read per million (RPM) of all genes that were considered to have expression variation above technical background variation (as estimated by spike-ins, see Methods) for all cells in three independent sequencing runs separated into control cells (Ctrl, Drosha Parental) and Drosha knock-out cells (Drosha KO).

File Name: Supplementary Data 2
Description: Lists of high confidence pair-wise gene expression covariations (see Methods) separated into positive and negatives covariations and into those observed in control cells (Ctrl, Drosha Parental) and those observed in Drosha knock-out cells (Drosha KO).

File Name: Supplementary Data 3
Description: Comprehensive list of all smFISH probes used for the detection of five selected genes
